# Formation of Aldehyde and Ketone Compounds during Production and Storage of Milk Powder

**DOI:** 10.3390/molecules17089900

**Published:** 2012-08-17

**Authors:** Yanhua Li, Lanwei Zhang, Weijun Wang

**Affiliations:** School of Food Science and Engineering, Harbin Institute of Technology, Harbin 150090, China; Email: liyanhuaid@yahoo.com.cn (Y.L.); wangweijunid@sohu.com (W.W.)

**Keywords:** aldehyde, ketone, milk powder, process, storage

## Abstract

Certain aldehyde and ketone compounds can be used as indicators, at a molecular level, of the oxidized flavor of milk powder instead of sensory evaluation. This study investigated the formation of aldehyde and ketone compounds as affected by the heat-related processing and storage of milk powder. The compounds were extracted by solid phase microextraction fiber and determined using gas chromatography-mass spectrometry. In the results, higher contents of hexanal, 2-heptanone, octanal and 3-octen-2-one were detected in concentrated milk and fresh milk powders than in raw milk and heated milk. The levels of these compounds increased with increasing time of storage of milk powder. Meanwhile, the DPPH radical scavenging activity decreased and peroxide value increased during the production and storage of milk powder. In addition, the pore volume distribution of milk powder particle was determined by nitrogen isotherm adsorption. The porosity of milk powder was significantly correlated to the changes of aldehyde and ketone compounds during storages periods of 3 months (r > 0.689, *p *< 0.05) and 6 months (r > 0.806, *p* < 0.01). Therefore attention should be paid to the detectable aldehyde and ketone molecules to control the oxidized flavor, which was influenced by pre-heating as well as concentration and drying during milk powder production.

## 1. Introduction

Milk powder is an important product for both direct consumption and as a food ingredient. The shelf life has been reported as anywhere from 3 months [1] to 3 years [2], while off-flavor formation for whole and skim milk powders occurs as quickly as 3–6 months [1,3]. It is well known that the quality of stored milk powder will not be exactly the same as fresh milk powder, but the differences should not be large enough that consumers find this stored milk powder unacceptable within the projected shelf life. The whole milk powder industry is, however, from time to time confronted by situations where powders demonstrate lower flavor stability than expected [4].

Flavor profiles of whole milk powder can be influenced by animal feed [5], season [3,6], processing operations [7,8] and storage conditions, including water activity, moisture, packaging, light and temperature [9,10,11]. Many different classes of volatile compounds are produced during the manufacture and storage of whole milk powder and most are formed via lipid oxidation and Maillard reactions. Certain compounds, such as aldehydes, ketones and lactones, contribute to the oxidized flavor in dairy products being variously described as being grassy, soapy, metallic, cardboardy, tallowy or fishy flavor. The aldehyde and ketone molecules can be detected instrumentally, and are of concern from a flavor as well as a safety standpoint [3,12].

Most studies on milk flavor have focused on the thermal treatment processes of milk, such as pasteurization, ultra-high temperature and sterilization. The contents of several ketones, aldehydes and sulphur compounds have been reported to increase during the processing and storage period [3,9,13,14], but comprehensive information is lacking on the aldehyde and ketone compounds and their changes during the production and storage of whole milk powder. Stapelfeldt *et al.* [7] found that levels of free radicals and thiobarbituric acid reactive substances of low-heat whole milk powder were higher than the levels in high-heat and medium-heat milk powder and the sensory quality of low-heat powder dropped to an unacceptable level within 33 days of storage at 45 °C. The research suggested that heat treatment could affect the oxidized status and flavor of milk powder.

Pre-heating, concentration and spray-drying are heat-related processes during the whole milk powder manufacture. The pre-heating step has been considered as the one of greatest importance for controlling the technological characteristics of the final product [15]. However the research on the effects of concentration and drying is very limited. The aims of this study were to: (1) determine the effects of heat-related processes and storage of whole milk powder on the levels of aldehyde and ketone compounds and oxidized status, and (2) evaluate the relationships between the pore characteristics of whole milk powder particles and the formation of these compounds during storage.

## 2. Results and Discussion

### 2.1. Milk Components Analysis

The production of milk powder was repeated using the raw milk from the same plant with two day intervals. There is no significant difference between the compositions of raw milk from different batches. Also, no differences were found in the compositions of different milk powders (*p* > 0.05). The average values of the components in all the milk powders were as follows: humidity, 3.5 ± 0.3%; protein, 26.5 ± 2.3%; fat, 25.7 ± 3.7%.

### 2.2. Changes in Aldehyde and Ketone Compounds

#### 2.2.1. Production of Milk Powder

The volatile compounds detected in raw milk (RM), heated milk (HM), concentrated milk and milk powders by solid phase microextraction (SPME)-gas chromatography (GC)-mass spectrometry (MS) mainly included straight chain aldehydes (hexanal, heptanal, octanal and nonanal), methyl ketones (2-heptanone and 2-nonanone), ketenes (3-octen-2-one and 3,5-octadien-2-one) and olefinic aldehydes (2-nonenal and 2-decenal).

These compounds have been previously documented in milk powder and were associated with different oxidized flavors [1,3,9,16]. Four compounds, hexanal, 2-heptanone, octanal and 3-octen-2-one were selected to discuss the changes in oxidized flavor during the heat-related processes, *i.e.*, pre-heating, concentration and drying, and subsequent storage of milk powder. The four volatile compounds were responsible for green grass, soapy/spicy, citrus/green and mushroom oxidized-odors in dairy products, respectively.

**Figure 1:**
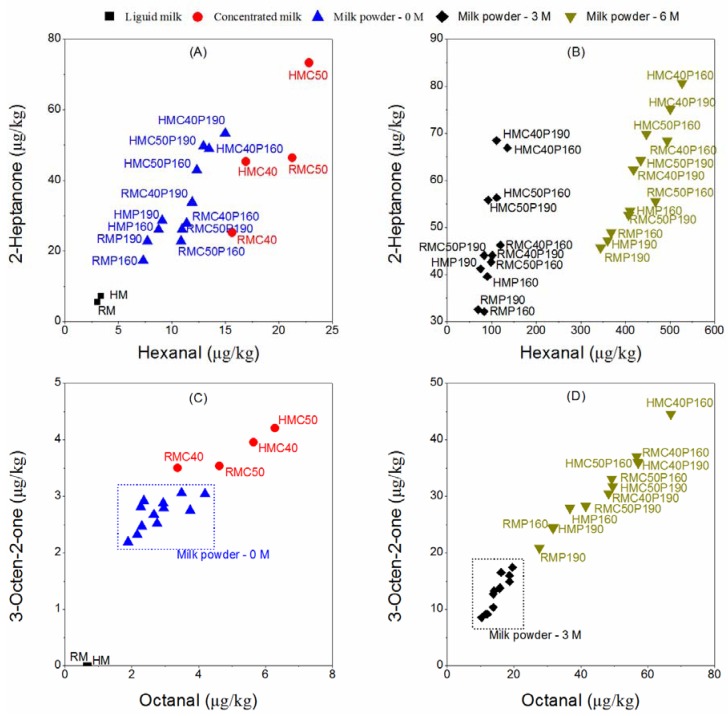
Changes in volatile compounds of milk. (**A**) Hexanal and 2-heptanone in different processing conditions of milk during the production of milk powder and (**B**) their differences on 3 and 6 months of storage; (**C**) Octanal and 3-octen-2-one in different processing milk during the produce of milk powder and (**D**) their differences on 3 and 6 months of storage. RM: raw milk; HM: heated milk; RMC and HMC: the concentrated milk of RM and HM; RMP, HMP, RMCP and HMCP: the milk powder of RM, HM, RMC and HMC; 40 and 50: concentration temperature (°C); 160 and 190: drying temperature (°C); 0 M, 3 M and 6 M: storage time (months).

As shown in Figure 1, levels of aldehyde and ketone compounds in liquid milk (RM and HM) were found to be lower than those in concentrated milk and milk powders. The contents of hexanal, 2-heptanone and octanal in RM was only 3.03, 5.44 and 0.71 μg/kg respectively, and 3-octen-2-one was not even detected. Hexanal, octanal and 2-heptanone could be considered as the flavor impacting compounds in RM [17]. These compounds in HM had no significant difference with RM (*p* > 0.05). However, the compounds in concentrated milk and milk powders in the heated groups (HMC, HMP and HMCP) had higher levels than the corresponding unheated groups (RMC, RMP and RMCP). This finding indicated that the pre-heating treatment could accelerate the formation of aldehyde and ketone compounds in concentrated milk and milk powders.

Concentrated milk had higher levels of hexanal, octanal and 3-octen-2-one than newly produced milk powders (Figure 1A,C) according to changes in the means of data, particularly HMC had significant higher contents of these three compounds than the corresponding newly produced milk powder (*p* < 0.05). This result may be due to the loss of flavor during drying and the difference in flavor release between the reconstituted milk powder and the concentrated milk. The aldehyde and ketone compounds in concentrated milk produced at 50 °C had higher levels than at 40 °C, although only hexanal and 2-heptanone showed significant differences (*p* < 0.05). The four compounds in concentrated milk were higher than those in RM and HM (*p* < 0.05), as shown in Figure 1A,C. The data suggested that the lipid oxidation continued under the concentration conditions, *i.e.*, low temperature and reduced pressure.

#### 2.2.2. Storage of Milk Powder

Great differences were observed in the aldehyde and ketone compounds of stored milk powder, as shown in Figure 1B,D. The impact of a compound depends on its content and sensory threshold. Odor activity value (OAV), the rate of concentration and sensory threshold, is usually used to measure this impact. OAV of aldehyde and ketone compounds in milk powders were far more than 1 (data not shown), suggesting that these compounds could be important contributors to the flavor of milk powder. High levels of these compounds were generated within 3 months and increased significantly with increasing the storage life (*p *< 0.05). The stored milk powders had higher levels of aldehyde and ketone compounds when the concentration temperature was 40 °C than when concentration temperature was 50 °C. Moreover, the milk powders had higher contents of these compounds when dried at 160 °C than when dried at 190 °C, regardless of storage (Figure 1B,D). These changes in aldehyde and ketone compounds indicated that the processes of concentration and drying could influence the flavor formation during storage of milk powder.

The aldehyde and ketone compounds can be generated from autoxidation reactions of the unsaturated fatty acids of milk. During the production and storage of milk powder, hexanal and 3-octen-2-one could originate from linoleic acid, while octanal could originate from oleic acid by autoxidation The spontaneous decomposition of hydroperoxides could also promote the formation of these compounds [18,19]. 2-Heptanone, a main methyl ketone in dairy products, is formed by β-oxidation of saturated fatty acids followed by decarboxylation or by decarboxylation of β-ketoacids naturally present in milk fat [20]. In the stored milk powders, hexanal had the largest increase among four volatile compounds as compared with the newly produced milk powders, and therefore hexanal might be the main indicator of oxidized flavor in milk powder.

### 2.3. Changes in Oxidative Status of Milk

#### 2.3.1. Antioxidative Activity

**Figure 2:**
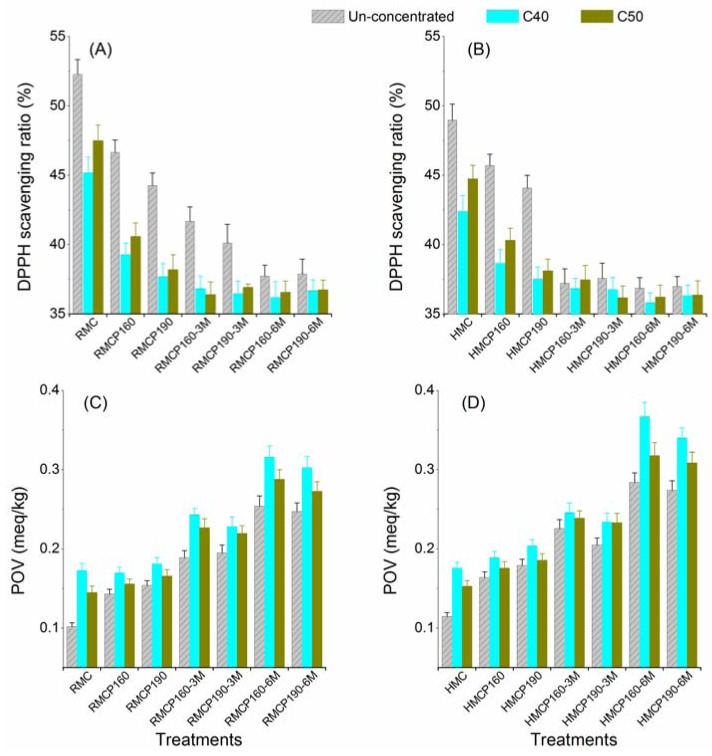
DPPH scavenging ratio (**A** and **B**) and peroxide value (**C** and **D**) of milk. RM: raw milk; HM: heated milk; RMC and HMC: the concentrated milk of RM and HM; RMCP and HMCP: the milk powder of RMC and HMC; C40 and C50: concentration temperature (°C); 160 and 190: drying temperature (°C); 3 M and 6 M: storage time (months).

As shown in Figure 2, the diphenyl picrylhydrazyl (DPPH) scavenging ratio and the peroxide value (POV) were investigated for measuring lipid oxidative stability of milk samples. RM (52.26%) showed a higher DPPH scavenging ratio than HM (48.97%). The ratio further decreased after the processes of concentration or drying. The DPPH scavenging ratio of concentrated milk or un-concentrated milk powders (RMP and HMP) was lower than HM and higher than the concentrated milk powders (RMCP and HMCP). The storage of milk powders did not significantly affect the DPPH scavenging ratio, except of RMP, which probably suggested there were hardly any antioxidative resources left in most milk powders.

The total antioxidative activity of whole milk could be due to the contribution of individual antioxidants, such as ascorbic acid, α-tocopherol, glutathione peroxidase and casein and lactoferrin occurring in whey [21,22]. The decrease in DPPH scavenging ratio indicated the total antioxidative activity (without distinguishing the contributions from individual compounds) decreased gradually after each single processing step.

#### 2.3.2. Peroxide Value

The oxidative stability of dairy products is the result of a delicate balance between the anti- and pro-oxidative processes in milk. POV of milk could be used to evaluate the oxidative status of milk samples [21]. Figure 2C,D show the changes in POV between liquid milk, concentrated milk and milk powders. RM and HM showed a lower POV than concentrated milk and milk powders. The POV of concentrated milk and milk powders ranged from 0.145–0.176 meq/kg and 0.143–0.367 meq/kg. Especially, the milk powders after 3 months’ storage showed a steep increase of POV. The oxidative status of milk powders was affected by the pre-heating because of the higher POV in the HM group (Figure 2D) than RM group (Figure 2C). The concentration also promoted the peroxide formation during the storage of milk powder. Taking storage for 6 months as an example, the milk powders that were concentrated at 40 °C had higher POV than those concentrated at 50 °C and the un-concentrated samples.

The prooxidant activity in milk depends on the presence of reactive oxidants and the antioxidant defense capacity. The formation of radicals, decrease of total antioxidative activiy, production of peroxides and oxidation of unsaturated lipids play important roles in lipid oxidation [22,23,24]. According to the volatile compound results (Figure 1), the changes in the total antioxidative activity and in the peroxide formation might be responsible for the differences in oxidized flavor due to different processes, although decarboxylated β-keto acids and oxidated saturated fatty acids might also contribute to the formation of methyl ketones.

### 2.4. Pore Characteristic of Milk Powder Particles

**Figure 3:**
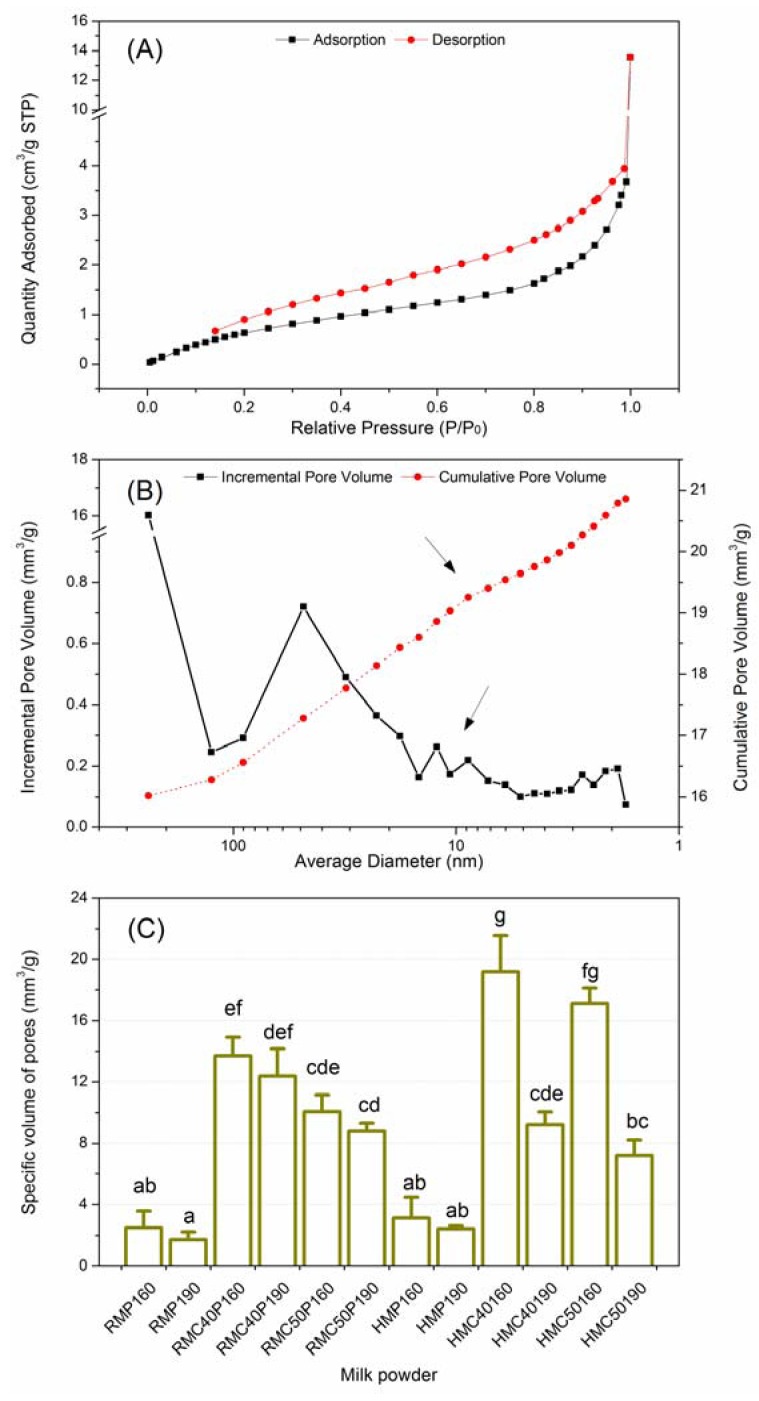
Pore distribution of milk powder. (**A**) Typical adsorption-desorption isotherm plot of milk powder; (**B**) Typical pore volume distribution functions as pore size of milk powder particles; (**C**) The difference in porosity of different milk powder. The different letters on the columns were significantly different among the treatments (*p* < 0.05). RM: raw milk; HM: heated milk; RMC and HMC: the concentrated milk of RM and HM; RMP, HMP, RMCP and HMCP: the milk powder of RM, HM, RMC and HMC; 40 and 50: concentration temperature (°C); 160 and 190: drying temperature (°C).

Figure 3A shows a typical adsorption-desorption isotherm plot of milk powder. With the increasing relative pressure (P/P_0_), nitrogen was gradually being absorbed in the pores of milk powder. A slight concave curve was seen in in low P/P_0_ range, while the intermediate zone was relatively flat. The curve in the higher P/P_0_ range raised rapidly, and a large amount of adsorption occurred when the relative pressure was close to 1. Comparing adsorption to desorption, hysteresis was found in the adsorption curve suggesting that the sizes of pores might change between adsorption and desorption.

Pore distribution of milk powder was determined in the range of pore size from 1.7 nm to 300 nm. Figure 3B shows the pore volume distribution of milk powder particles with the average pore size distribution. The pore size and volume of milk powder particles were different and had a continuous distribution. The large increase of volume was found in the range of ~50 nm of pore size. The cumulative volume appeared to have an accelerated upward trend in the range of ~10 nm (arrow), indicating that small pores could also contribute to the cumulative volume as the large pores did. 

Differences in average porosity (volume per gram) were found among the different milk powders, as shown in Figure 3C. The porosity of milk powders had a range of 1.73–17.09 mm^3^/g. With univariate ANOVA tests of certain parameters (porosity by pre-heating, concentration and drying processes), we found that the processes significantly influencing the porosity were the concentration (*p* < 0.001), the drying (*p* < 0.05) and the heating (*p* < 0.05). The heat-related processes, besides pre-heating should therefore be considered to classify the types of milk powder, because concentration and drying also affected milk powder particles, resulting in changes to the final products, such as the oxidized flavor during storage.

The static nitrogen adsorption method has been widely used in porosity analysis of food matrices. Using the BJH (Barrett-Joyner-Halenda) model, the average pore diameter of milk powders had a range of 5.4–27.8 nm, and the pores belonged to the mesopore (2–50 nm) class according to the International Union of Pure and Applied Chemistry (IUPAC) definition. Milk powder particles are produced instantly when droplets contact dry hot air. These particles inevitably adsorb oxygen, which may increase the risk of oxidation in milk powder. In the paper, porosity values were significantly correlated to the selected volatile compounds of milk powders during storages of 3 months (r > 0.689, *p* < 0.05) and 6 months (r > 0.806, *p* < 0.01). The heat-related processes in the manufacture of milk powder affected the porosity of milk powder particles. It is curious the aldehyde and ketone compounds in concentrated milk produced at 50 °C had higher levels than at 40 °C according to changes in the means of data, while the stored milk powders had higher levels of aldehyde and ketone compounds when the concentration temperature was 40 °C than when concentration temperature was 50 °C. The effect of temperature of concentration this has opposite effects in concentrates and powders. The reason might be that the milk powder had large porosity when the concentration temperature was 40 °C. Obviously, the larger the porosity of the milk powder particles, the more easily oxidized volatiles were formed. In the study, the larger porosity of milk powder was found when it was produced at 160 °C than those produced at 190 °C, although only HMCP showed a significant (*p* < 0.05) difference. During spray-drying the speed of shrinking of milk droplets could affect the surface composition of milk powder particles, e.g., the free fat [25,26] which could promote the formation of oxidized compounds. Therefore, the flavor stability of milk powder could be improved by controlling these processes.

## 3. Experimental

### 3.1. Laboratory-Scale Manufacture of Milk Powder

RM was obtained from a local dairy plant and 0.02% (w/v) NaN_3_ was added to prevent bacterial growth. HM was prepared immediately according to the method of Lee and Sherbon [27] using a temperature of 90 °C for 30 s. The milk was cooled to ambient temperature (20 ± 1 °C) in a stirred water bath at 0–4 °C and prepared for concentration and drying. The concentration was performed according to the method of Drohan *et al*. using a rotary evaporator (N-1000, EYELA, Tokyo, Japan) [28]. RM (and HM) was concentrated to four times at 40 °C and concentrated three times at 50 °C, respectively, under 0.1 MPa, and the concentrated milks were subclassified according to the concentration temperature used. Spray-drying for RM, HM and the concentrated milk of RM and HM (RMC and HMC) was operated using a laboratory-scale spray dryer (SD-1000, EYELA). The RM, HM, RMC and HMC milk powders (RMP, HMP, RMCP and HMCP) were subclassified according to the inlet-air temperature (160 and 190 °C). Other parameters were as follows: nozzle pressure, 20 MPa; feed flow rate, 400–600 mL/h; flow rate of drying air, 40–60 m^3^/h. The outlet-air temperature was controlled at 85 °C by adjusting the speed of material and hot air. In total, RM, HM, four concentrated milk and twelve milk powder samples were collected for analysis. Milk powders were sealed in plastic bags and stored in a dry and air-tight container at ambient temperature (20 ± 1 °C) for 3 and 6 months. 

The concentrated milk and milk powder were reconstituted according to the total solids by adding Milli-Q water (Millipore Corporation, Billerica, MA, USA) at ambient temperature. Two kinds of reconstituted milk were prepared by stirring the samples with a stirrer (JJ-1, Guohua Inc., Jiangshu, China) at 100 rpm for 5 min, and a 2-hour equilibration period was used before measurements. Total solids were determined by drying 10 g of the sample at 105 ± 1 °C [29]. Milk fat was determined by the Rose-Gottlieb method and total protein by the Kjeldahl technique with a factor of 6.38 as described by Guinee *et al*. [30]. 

### 3.2. Extraction of Volatile Compounds

The volatile compounds in the headspace of samples were obtained using SPME with a divinylbenzene/carboxen/polydimethylsiloxane fiber (2 cm length, Supelco Inc., Bellefonte, PA, USA). In details, 150 mL sample (RM, HM, distillate, concentrated milk and reconstituted milk powders) was weighed into a 250 mL vial and 60 μL aqueous solution of 50 mg/kg 4-methyl-2-pentanone (Sigma, St. Louis, MO, USA) were added as internal standard. The sample was equilibrated at 55 °C for 45 min under mild stirring with a magnetic stirrer (HJ-3, Guohua Inc., Jiangshu, China), and then extracted under the same conditions as the fiber.

### 3.3. Determination of Volatile Compounds

The SPME fiber was desorbed for 2 min in the injector of GC before analysis. The mass spectra of compounds were obtained using the HP 6890 GC equipped with the HP 5973 mass selective detector (Hewlett-Packard Inc., Wilmington, DE, USA). The interface, quadrupole and ion source temperature were kept at 250, 280 and 230 °C respectively. A DB-5 capillary column with 60 m length, 0.25 mm internal diameter, 0.25 μm phase thickness (J&W Scientific, Folsom, CA, USA) was used. The oven temperature was maintained at 40 °C for 8 min, programmed to 150 °C at a rate of 4 °C/min, then raised at 20 °C/min to 250 °C and held at 250 °C for 5 min. Helium was used as carrier gas at a flow rate of 1.0 mL/min. Electron impact ionization of MS was used at a voltage of 70 eV. The mass range was *m*/*z *30–500. The selected compounds (hexanal, 2-heptanone, octanal and 3-octen-2-one) were identified using the the NIST-02L GC-MS spectrum library and the retention time of their respective standards. The contents of individual compounds were calculated using the area ratio of flavor compounds and internal standard. 

### 3.4. DPPH Radicals Scavenging Activity

The total antioxidative activity was determined by the DPPH method [21,31] with some modification. 2,2-Diphenyl-1-picrylhydrazyl solution (200 μmol/L) in absolute methanol was employed. Milk samples were mixed vigorously with DPPH solution (1:3, v/v) for 15 s. The mixture was centrifuged at 5,000 *g* for 10 min. The supernatant was held at 20 °C for 20 min in a dark place.

### 3.5. Peroxide Value

POV was used to analyze the oxidation of milk. POV analysis was based on the procedure of Smet *et al*. [32].

### 3.6. Nitrogen Adsorption Analysis of Milk Powder

Nitrogen adsorption-desorption isotherms were obtained using an automatic adsorptiometer (Micromeritics, ASAP 2020, Norcross, GA, USA). The sample was equilibrated at 75 °C and 10^−2^ Pa for 1 h. BJH method was used to calculate the pore size distribution from nitrogen adsorption-desorption isotherms obtained at 77.2 K.

### 3.7. Statistical Analysis

Experiments were triplicated for manufactures and detections of milk powders. The results were statistically analyzed using PASWStatistics18.0 Software (SPSS Inc., Chicago, IL, USA). One-way and univariate analysis of variance (ANOVA) were applied to examine the effects of processes. Pearson’s correlation (2-tailed) was carried out to analyze the relationships of DPPH scavenging ratio and porosity with 4 selected volatile compounds. 

## 4. Conclusions

The results of this study indicated that the pre-heating could improve the formation of volatile compounds in concentrated milk and milk powders. The processes of concentration and drying could influence the stability of these compounds during storage. Decrease of antioxidative activity and increase of POV during the production and storage suggested that there was hardly any antioxidative resources left in most of the milk powder samples. The POV results after 3 months storage of milk powders showed that pre-heating and concentration could affect the peroxide formation during storage. Moreover, the heat-related processes could influence the porosity of milk powder. Therefore, attention should be paid to the concentration and drying, as well as the pre-heating, during the manufacture and storage of milk powder due to their effects on the oxidized flavor-related volatile compounds.
